# Integrating acute malnutrition interventions into national health systems: lessons from Niger

**DOI:** 10.1186/s12889-016-2903-6

**Published:** 2016-03-10

**Authors:** Hedwig Deconinck, Mahaman Hallarou, Bart Criel, Philippe Donnen, Jean Macq

**Affiliations:** Institut de recherche santé et société, Université catholique de Louvain, Clos Chapelle-aux-Champs 30, Brussels, 1200 Belgium; Ecole de santé publique, Université libre de Bruxelles, route de Lennik 808, Brussels, 1070 Belgium; Institute of Tropical Medicine, 155 Nationalestraat, Antwerp, 2000 Belgium

**Keywords:** Acute malnutrition, Integration, Indicators, Health system, Low-income countries, Niger

## Abstract

**Background:**

Since 2007, integrated care of acute malnutrition has been promoted in Niger, a country affected by high burden of disease. This policy change aimed at strengthening capacity and ownership to manage the condition. Integration was neither defined nor planned but assumed to have been achieved. This paper studied the level and progress of integration of acute malnutrition interventions into key health system functions.

**Methods:**

The qualitative study method involved literature searches on acute malnutrition interventions for children under 5 in low-income countries to develop a matrix of integration. Integration indicators defined three levels of integration of acute malnutrition interventions into health system functions—full, partial or none. Indicators of health services and health status were added to describe health system improvements. Data from qualitative and quantitative studies conducted in Niger between 2007 and 2013 were used to measure the indicators for the years under study.

**Results:**

Results showed a mosaic of integration levels across key health system functions. Four indicators showed full integration, 22 showed partial integration and three showed no integration. Two-thirds of system functions showed progress in assimilating acute malnutrition interventions, while six persistently stagnated over time. There was variation within and across health system domains, with governance and health information functions scoring highest and financing lowest. Steady improvements were noted in geographic coverage, access and under-5 mortality risk.

**Conclusions:**

This study provided useful information to inform policy makers and guide strategic planning to improve integration of acute malnutrition interventions in Niger. The proposed method of assessing the extent of integration and monitoring progress may be adapted and used in Niger and other low-income countries that are integrating or intending to integrate acute malnutrition interventions.

**Electronic supplementary material:**

The online version of this article (doi:10.1186/s12889-016-2903-6) contains supplementary material, which is available to authorized users.

## Background

During the emergency response to the 2005 food crisis, technical and financial partners of the Ministry of Public Health (MOH) of Niger introduced a new approach to address acute malnutrition. This approach treated most children with the condition as outpatients in decentralised care rather than hospitalising them, and added active community case finding for early start of treatment. The demonstrated effectiveness and feasibility of the new approach prompted the MOH to issue directives in 2007 for integrating acute malnutrition interventions into public health care. From then onward, partners no longer implemented acute malnutrition care but instead progressively provided support to the government for implementation. The policy change aimed at government ownership and scale-up the intervention by strengthening national and local capacity to improve access and coverage for the 1 million children under 5 years of age affected with acute malnutrition every year in Niger [1].

Despite the countrywide decentralisation of the management of acute malnutrition, less than one-third of all children with acute malnutrition were admitted for treatment in 2013 [2]. As in most low-income countries with a high burden of acute malnutrition, Niger’s health system faced important operational capacity and resource challenges to delivering acute malnutrition interventions. Evaluations of acute malnutrition programmes conducted in low-income countries between 2007 and 2013 [3–5] identified challenges in systemic, organisational and clinical capacities for quality of care, integration and scale-up. With continuing high caseloads of acute malnutrition in Niger, time and again, questions about how to improve access and coverage of the integrated interventions surfaced. One systematic review studied the level of integration of health worker programmes into health systems [6], but no such studies on level of integration have been published on acute malnutrition. This paper assessed the level and trend of integration of acute malnutrition interventions in Niger since 2007. Factors that influenced the integration process were discussed in another paper. The results of the assessment may be useful to inform policy makers and guide strategic planning for improved acute malnutrition services.

## Methods

We applied qualitative methods to study the status of integration of acute malnutrition interventions in Niger. For the purpose of this paper, integration was defined from a health system perspective as the extent, pattern and rate of adoption and assimilation of interventions for management of acute malnutrition into key functions of the national health system [7]. Integrated care of children with acute malnutrition and other illness was central to our study and viewed as a result of a process and activities undertaken at multiple levels of systemic, managerial, organisational, professional and clinical integration [8].

The study methods consisted of three distinct steps. First, we reviewed published and grey literature on acute malnutrition interventions and their integration into national health systems [9]. We then adapted a list of key health system functions for the integration of acute malnutrition interventions from a framework that had been used and validated in different countries [4, 9]. Functions were organised according to the six domains of the health system [10] (i.e. governance, health financing, health information, health workforce, medical products and service delivery). We then developed a diagnostic tool with 29 indicators to measure past and current integration of acute malnutrition interventions into the identified key health system functions. Guided by the literature [6, 7, 11], we defined the integration indicators and their level of integration along a continuum (no interactions or segregation when no integration existed; linkage or coordination when partial integration existed; and full integration when health system functions and structures had assimilated or merged [12]). The diagnostic tool also included indicators for measuring the performance (geographic coverage, access to treatment, quality of care, contact coverage and sustainability) of acute malnutrition services and two indicators for measuring health status (prevalence of acute malnutrition and under-5 mortality). Indicators and their levels are described in the Additional file 1: Table S1. Second, we used data from qualitative and quantitative studies conducted in 2007 [13], 2010 [14] and 2013 [15] to measure integration and performance of acute malnutrition services and health outcome [1, 16–20] as proposed by the indicators. The qualitative studies reviewed health system capacity to implement and integrate acute malnutrition interventions in health districts in Niger between June 2007 and March 2014. The respective study teams, lead by the first author, visited national, regional and district hospitals, health centres, health posts and communities in seven out of eight regions (Diffa, Dosso, Maradi, Niamey, Tahoua, Tillabéry and Zinder). Data were collected through key informant interviews and semi-structured focus group discussions with government administrators and partners at national, regional, district and community level, district health managers and health workers, community volunteers and members and service users. Level-adapted questionnaires with open-ended questions were developed and tested before use. The working language was French, and simultaneous translation was available for discussions in the local languages. Findings were recorded on paper, summarized in a spreadsheet, analysed by theme, triangulated and summarized [13–15]. In addition, relevant policies, implementation strategies, guidelines and annual action plans at national, regional and district levels, as well as national, regional and district databases were consulted to assess the quality of planning and performance of service delivery. Third, participation in national coordination meetings and planning workshops allowed triangulation and validation of the proposed indicators definitions and levels and their results.

## Results and discussion

This qualitative study assessed the level of integration of acute malnutrition interventions into key health system functions in Niger and mapped the progress of integration for 2007, 2010 and 2013 (Table 1). Results showed a mosaic of integration levels across health system functions. Four indicators showed full integration, 21 showed partial integration and three showed no integration. Two-thirds of system functions showed progress in assimilating acute malnutrition interventions, and six persistently stagnated over time. There was variation within and across domains. Governance and health information system functions scored best, and health financing scored worst. The acute malnutrition services indicators of geographic coverage and access to treatment improved, the indicator of quality of care was adequate and stable, and the indicators of contact coverage and sustainability remained low. The health status indicator of acute malnutrition prevalence was unstable for the years under study but showed improvement for 2013, and of under-5 mortality risk showed improvement for 2013.

**Table 1:** Integration level of acute malnutrition interventions and health outcome in Niger

Elements	Indicator	2007	2010	2013
Health system functions				
Governance				
Policy setting	National health and nutrition policies with the integrated management of acute malnutrition (IMAM) as part of child health care (i.e. integrated management of childhood illness (IMCI) and child hospital care)	No	Partial	Full
National guidelines	National guidelines for IMAM supporting comprehensive child health care	No	Partial	Full
Technical leadership	A technical advisory group for comprehensive child health care	Partial	Partial	Partial
Regulation and coordination	Regulation and coordination of health actors (including financial and technical partners, education and training institutions, professional associations, private and informal health sector, communities and champions) aligning with the national health and nutrition policy and implementation strategy	Partial	Partial	Partial
Social participation	Social participation of local and community actors in planning, implementing and monitoring child health care with a people-centred care approach	No	No	No
Health financing				
Regular budget-pooled funding	Regular budget from pooled funds with a sector-wide approach covering financing for IMAM	No	No	Partial
Annual costed action plans	Annual costed action plans of MOH covering IMAM interventions	No	No	Partial
Health workers payroll	Staff in national health facilities involved in IMAM on MOH payroll	No	No	Partial
Financial risk protection	Fee waiver system for children under 5 covering comprehensive child health care	No	Partial	Partial
Health information				
Health information system (HIS)	National HIS, including acute malnutrition indicators	No	Partial	Full
Performance monitoring system	Performance monitoring of comprehensive child health care	No	Partial	Full
Contact coverage monitoring	IMAM coverage monitoring as part of child health care coverage monitoring	No	No	No
Health workforce				
Adequate coverage of health workers	Adequate number of qualified health workers with geographic coverage for comprehensive child health care	No	Partial	Partial
Competences of health managers and health workers	Adequate technical and organizational management skills for comprehensive child health care	Partial	Partial	Partial
Performance appraisal and motivation system	Performance appraisal and career development opportunities as part of the human resources management system	No	Partial	Partial
Pre-service education	Modules of pre-service education curriculum on comprehensive child health and nutrition	No	Partial	Partial
Continuing professional development	Continuing professional development on comprehensive child health and nutrition	No	Partial	Partial
Medical products				
Essential medicines and medical supplies list	National essential drugs and medical supplies list, including for IMAM	No	Partial	Partial
Procurement system	National drugs and medical supply needs (forecasting and) procurement including for IMAM	No	No	Partial
Logistic management system	National logistic management system for drugs and medical supplies including for IMAM	No	Partial	Partial
Service delivery				
Demand generation	Demand generation by activating and informing communities for improved child health and nutrition	No	No	No
Early case finding	Early active (by volunteers in the community), systematic (by health workers at the health facility) and enhanced (by carer) case finding for selected child illnesses	No	Partial	Partial
Community-based primary care	Promotive and preventive community health and nutrition (and community case management)	No	Partial	Partial
Outpatient care (facility-based primary care)	Outpatient management of severe acute malnutrition (SAM) without complications and moderate acute malnutrition (MAM) as part of IMCI	No	Partial	Partial
Inpatient care (child hospital care)	Inpatient management of SAM with complications until stabilisation as part of child hospital care	No	Partial	Partial
Health outreach	Health outreach activities for selected child illnesses, including acute malnutrition	No	Partial	Partial
Referral and tracing between services	Referral and tracing system for detection and retention in treatment of selected child illnesses, including acute malnutrition	No	Partial	Partial
Patient-centred continuity of care	Comprehensive child health care tracked over time and place responding to individual preferences, needs and values	No	No	Partial
Continuous quality improvement	Continuous quality improvement of comprehensive child health care	No	Partial	Partial
Health services performance				
Geographic service coverage	Number of sites with SAM services	30 [13] (<5 %)	772 [26] (>70 %)	944 [15] (>95 %)
	(Proportion (%) of primary health facilities offering SAM services)			
	Number of sites with MAM services	610 [13] (<5 %)	850 [26] (>75 %)	1 180 [15] (>95 %)
	(Proportion (%) of primary health facilities offering MAM services)			
Access to treatment	Annual number of children under 5 accessing treatment for SAM	60 843 [13]	330 000 [26]	406 327 [15]
	Annual number of children under 5 accessing treatment for MAM	275 030 [13]	257 000 [26]	520 398 [15]
Quality of care	Annual overall SAM cure, case-fatality and defaulting rates	Good [13]	Good [26]	Good [15]
	Annual overall MAM cure, case-fatality and defaulting rates	Good [13]	Good [26]	Good [15]
Contact coverage	Proportion of children under 5 diagnosed with SAM in the population receiving treatment	Low [13]	Low [27]	Low [15]
Sustainability	Sustainability based on financial and technical dependence of IMAM interventions	Low [13] (NA)	Low [14] (41 %; 13)	Low [15] (60 %; 20)
	(Proportion (%) of primary health facilities with IMAM services receiving technical partner support; number of technical partners)			
Health status				
Prevalence of acute malnutrition	Proportion (%) of children under 5 diagnosed with SAM in the population (confidence interval)	0.8 % [19] (NA)	3.2 % [17] (2.7–3.7)	2.6 % [1] (2.2–3.1)
	Proportion (%) of children under 5 diagnosed with overall acute malnutrition in the population (confidence interval)	11.0 % [19] (NA)	15.5 % [17] (14.2–16.8)	13.3 % [1] (12.3–14.3)
Under-5 mortality	Probability of dying before 5 years of age expressed per 1000 live births	230 [19] (217–244)	NA	127 [18] (119–136)
	Proportion of deaths of children under 5 in the population per 10000 children per day (confidence interval)	1.8 [20] (NA)	2.1 [17] (NA)	0.7 [1] (NA)

IMAM: Integrated Management of Acute Malnutrition; IMCI: Integrated Management of Childhood Illness; NA: not available; MAM: Moderate Acute Malnutrition; MOH: Ministry of Public Health; SAM: Severe Acute Malnutrition

The study findings that integration of acute malnutrition interventions were only partially achieved contrasted with the general perception in Niger that the management of acute malnutrition was integrated into the national health system. The following sections discuss what integrated management of acute malnutrition meant in Niger, what was expected of it and what can be learned from it.

### Understanding integration and its aim

Since the Niger MOH called for the provision of integrated management of acute malnutrition (IMAM) –*Prise en charge intégrée de la malnutrition aiguë–* as a routine health service in all public health facilities in 2007, it assumed instant integration. Integration initially was interpreted from an organisational perspective as care provided by the public health sector in all health facilities. With time, it evolved to mean integrated care of patients from a clinical perspective. The question of what “integrated care” means, or what dimension of integration is being addressed is common [8], and not just for Niger or the management of acute malnutrition. Integrative functions of primary care include dimensions of systemic, managerial, organisational and professional integration at the meso and macro level of population-based care that is needed to obtain clinical integration of patient-focused care at the micro level. Both functional and normative integration are integrative functions that cut across the different levels of care [12]). For Niger, the use of a clear taxonomy of integrative functions of care would contribute to the understanding of the complex and multi-dimensional nature of integrated care as a process and hence its improvement [8].

The quest of the MOH to integrate management of acute malnutrition was driven by its taking ownership of improving coordinated care as well as other assumptions. First, integration would rapidly scale up service delivery. Second, integration would resolve capacity concerns at national, regional and local governance and implementation levels. Third, integration would resolve all other concerns about quality of care, inequitable coverage, unbalanced resource allocations, and uncertain financial and institutional sustainability. A general belief associates integration of health services with improved efficiency, cost-effectiveness, client-orientation, equity, local ownership, quality and health status overall [21]. However, systematic reviews of integration of primary health care in low-income countries were unable to draw conclusions on the benefits and disadvantages of integrated services [21–23]. These studies have at least two relevant lessons for Niger. First, integrating a service does not necessarily mean integrating all of its components, as permutations are possible. Second, integration is not a cure for inadequate resources or capacities. For Niger, evidence of the effectiveness of integrative functions for improved acute malnutrition services and health outcome would inform appropriate strategy designs adapted to the country-context and the capacities of the MOH and its partners.

### Learning from the integration process

The mosaic pattern of integration of acute malnutrition interventions (Table 1) indicated a complex and dynamic process, or continuum [24] that showed gradual progress. The absence of a strategy or phased plan implied that integration resulted from a natural self-organisation rather than orderly planning. It also showed that achieving integrated care of children with acute malnutrition required changes not only in service delivery, but also in systemic and organizational dimensions of care. Furthermore, it showed that integration into key system functions did not progress simultaneously and therefore may not always be desirable, possible or opportune.

We identified two consequences of the failure to translate the 2007 policy on integrated management of acute malnutrition into an adapted implementation strategy. First, the policy set no goals for what aspects or components of acute malnutrition interventions to integrate how, when, why or where. Nor did it reflect on opportunities for and threats to integrated care of acute malnutrition or other health interventions. Because of the lack of a specific integration plan for IMAM in Niger, progress was not monitored and adverse or unintended effects on the health system were not studied or corrected. Positive effects of integration could have included extended coverage of interventions and increased capacity of the health system. Negative effects of integration could have included the removal of privileged status and the necessary pampering for maturation or the imbalanced care of public health priorities [25]. Second, the policy missed aligning health actors behind a common goal. As such, partners provided short-term support that yielded immediate results but did not collaboratively strengthen the health system with a long-term vision to adopt and assimilate integrated quality services sustainably. However, the policy was successful in the sense that partners gradually moved from direct implementation toward support for service delivery, which stimulated national and local ownership and political will and hence strengthened donor trust.

For Niger, an integration strategy for the management of acute malnutrition should set goals based on overall public health priorities and available capacities and resources. It should decide what aspects of interventions to integrate or not, and what aspects to outsource in the short or long term through, for example, public-private partnerships that are well regulated and coordinated as part of the same transformative change agenda. In some areas partners were well represented in the community and therefore well placed to implement the community-based interventions. Some partners were well placed to distribute bulky supplies to the periphery. Task sharing could be effective under an overarching and collaborative strategic plan with a defined goal and oversight from the MOH to regulate and coordinate activities.

The integration matrix proved useful to map the level of integration of IMAM into key system functions and show progress over time. In our study, the matrix was itself the outcome of a diagnostic of key heath system functions, but it should ideally be linked with an implementation strategy to be able to measure and interpret progress and inform policy adaptations. The proposed indicators matrix is therefore a diagnostic tool that itself may be used to inform strategies and formulate objectives for improved integrated care. It therefore needs to be adapted or changed depending on the changing country context, implementation strategy and level of integrative functions under study. We anticipate that lessons from this case study may inform the integration strategy of the multisectoral interventions of the national nutrition security policy under development in Niger.

The study had important limitations. Information was collected at three intervals. A separate paper investigated factors that influenced the process of integration of acute malnutrition interventions in Niger and complements the interpretation of the results presented here. Moreover, in the absence of a national strategy for integrating acute malnutrition interventions, progress could not be compared to national goals. Without knowing what to aim at, the pattern of integration as shown in the results matrix has limited use. The integration indicators are measured from a systemic perspective but mix types of integration (e.g. organisational, functional, professional) and levels of health system targeted (e.g. micro, meso, macro). Furthermore, the study was not designed to measure the effect of integration of acute malnutrition interventions on the overall health system, but we added indicators of performance of acute malnutrition services and health outcome to understand the context in which the integration process occurred. Nevertheless, we missed information on cost of a treatment, equity, extra-budgetary financial contributions or donor investments that played a role in improved quality and sustainability.

## Conclusions

The study developed and measured indicators to investigate the level and trend of integration of acute malnutrition interventions into key health system functions in Niger. The tool was able to assess the status and monitor the process of integration. The study uncovered the missed opportunity of a national integration strategy for integration of acute malnutrition interventions with clear goals and expectations that unlock local capacities and regulate partnerships for improved integrated care and sustainability.

We recommend that for future use, health actors consultatively and collaboratively adapt the indicator matrix to the integration strategy that is aligned with national health and nutrition policies and development plans and level of use. The proposed method of assessing and monitoring integration progress may be used in Niger and other countries to stimulate the debate of integrated care, and to improve strategic planning for improved health outcomes.

## Additional file

Additional file 1: Table S1. Indicators of integration level of acute malnutrition interventions and health outcome. (DOC 121 kb)

## Abbreviations

HIS: health information system
IMAM: integrated management of acute malnutrition
IMCI: integrated management of childhood illness
MAM: moderate acute malnutrition
MCH: maternal and child health
MOH: Ministry of Public Health
SAM: severe acute malnutrition
