# Supplementary material for: Integrating acute malnutrition interventions into national health systems: lessons from Niger
Source: BMC Public Health. 2016 Mar 10;16:249. doi: 10.1186/s12889-016-2903-6 (PMC4785683; doi:10.1186/s12889-016-2903-6)
Supplement: Additional file 1: Table S1. — Indicators of integration level of acute malnutrition interventions and health outcome. (DOC 121 kb) [file 12889_2016_2903_MOESM1_ESM.doc]

**Additional file 1: Table S1: Indicators of integration level of acute malnutrition interventions and health outcome**

| **Elements** | **Indicator** | **Level** |
| --- | --- | --- |
| **Health system functions** |  |  |
| **Governance** |  |  |
| Policy setting | National health and nutrition policies with the integrated management of acute malnutrition (IMAM) as part of child health care (i.e. the integrated management of childhood illness (IMCI) and child hospital care) | **Full:** National health and nutrition policies cover IMAM as part of IMCI and child hospital care.  **Partial:** National health and nutrition policies cover some but not all aspects of IMAM as part of IMCI and child hospital care.  **No:** National health and nutrition policies do not cover IMAM. |
| National guidelines | National guidelines for IMAM supporting comprehensive child health care | **Full:** National guidelines for IMAM exist and support comprehensive child health care.  **Partial:** National guidelines for IMAM exist but are ill adapted or incomplete for comprehensive child health care.  **No:** National guidelines for IMAM do not exist, or generic guidelines are used but are not adapted to the country context. |
| Technical leadership | A technical advisory group for comprehensive child health care | **Full:** The Ministry of Health (MOH) leads a technical advisory group for comprehensive child health care (with IMAM as part of IMCI and child hospital care) generating national technical and operational management expertise.  **Partial:** The MOH leads a technical advisory group for IMAM that is not part of an overarching child health care group.  **No:** A technical advisory group for IMAM does not exist, or exists but is not lead by the MOH. |
| Regulation and coordination | Regulation and coordination of health actors (including financial and technical partners, education and training institutions, professional associations, private and informal health sector, communities, and champions) aligning with the national health and nutrition policy and implementation strategy | **Full:** The MOH regulates and coordinates health actors to align with the national health and nutrition policy and implementation strategy with a common goal (with IMAM as part of IMCI and child hospital care).  **Partial:** Not all health actors involved in IMAM are regulated and coordinated by the MOH or aligned with the national health and nutrition policy and implementation strategy.  **No:** Health actors involved in IMAM are neither regulated nor coordinated by the MOH nor aligned with the national health and nutrition policy and implementation strategy. |
| Social participation | Social participation of local and community actors in planning, implementing and monitoring of child health care with a people-centred care approach | **Full:** The MOH involves local and community actors in planning, implementing and monitoring of child health care with a people-centred care approach.  **Partial:** The MOH involves local and community actors in some but not all aspects of planning, implementing and monitoring of child health, and may receive partner support for IMAM.  **No:** The MOH does not, but partners may involve local and community actors in planning, implementing and monitoring IMAM. |
| **Health financing** |  |  |
| Regular budget-pooled funding | Regular budget from pooled funds with a sector-wide approach covering financing for IMAM | **Full:** Financing for IMAM interventions is covered by the regular health budget from pooled funds with a sector-wide approach.  **Partial:** Financing for IMAM interventions is predominantly from extra-budgetary funds (short term or emergency funding).  **No:** Financing for IMAM interventions is from extra-budgetary funds (short term or emergency funding). |
| Annual costed action plans | Annual costed action plans of MOH covering IMAM interventions | **Full:** IMAM interventions are fully incorporated in annual costed action plans of MOH.  **Partial:** Some but not all IMAM interventions are incorporated in annual costed action plans of MOH.  **No:** IMAM interventions are not incorporated in annual action plans of MOH and thus have no health budget allocation. |
| Health workers payroll | Staff in national health facilities involved in IMAM on MOH payroll | **Full:** IMAM staff in national health facilities is on the MOH payroll.  **Partial:** Some but not all IMAM staff in national health facilities is on the MOH payroll, and some may have temporary local government or partner contracts.  **No:** No IMAM staff is on the MOH payroll. |
| Financial risk protection | Fee waiver system for children under 5 covering comprehensive child health care | **Full:** The MOH has a functional fee waiver system for children under 5 covering comprehensive child health care omitting all out-of-pocket payments.  **Partial:** The MOH has a fee waiver system for children under 5 that covers some but not all costs of IMAM, or does not omit all out-of-pocket payments.  **No:** No MOH fee waiver system for children under 5 exists, but partners may cover IMAM costs. |
| **Health information** |  |  |
| Health information system (HIS) | National HIS including acute malnutrition indicators | **Full:** The national HIS includes key acute malnutrition indicators.  **Partial:** The MOH manages an IMAM information system apart from the national HIS, and may receive partner support.  **No:** The MOH does not manage an IMAM information system. |
| Performance monitoring system | Performance monitoring of comprehensive child health care | **Full:** The MOH monitors performance of comprehensive child health care services including IMAM.  **Partial:** The MOH monitors some but not all aspects of child health care services including IMAM or of IMAM services only, and may receive partner support for IMAM.  **No:** The MOH does not, but partners may monitor IMAM service performance. |
| Contact coverage monitoring | IMAM coverage monitoring as part of child health care coverage monitoring | **Full:** The MOH monitors IMAM coverage as part of child health care coverage monitoring.  **Partial:** The MOH monitors IMAM-specific coverage, and may receive partner support.  **No:** The MOH does not, but partners may monitor IMAM-specific coverage. |
| **Health workforce** |  |  |
| Adequate coverage of health workers | Adequate number of qualified health workers with geographic coverage for comprehensive child health care | **Full:** The MOH manages shortage of qualified health workers for comprehensive child health care by hiring or re-allocating, training and retaining health workers and/or adjusting job descriptions for task sharing or shifting.  **Partial:** The MOH has shortage of qualified health workers for child health care that may be complemented by partner staff and/or by partner support for hiring for IMAM.  **No:** Shortage of health workers for IMAM is compensated for by partner staff. |
| Competences of health managers and health workers | Adequate technical and organizational management skills for comprehensive child health care | **Full:** The MOH staff has adequate technical and organizational management skills for comprehensive child health care.  **Partial:** The MOH staff has insufficient technical and organizational management skills for child health care, and may receive partner support for IMAM.  **No:** Partners are in charge of clinical and organizational management of IMAM. |
| Performance appraisal and motivation system | Performance appraisal and career development opportunities as part of the human resources management system | **Full:** The MOH provides performance appraisal and career development opportunities as part of its human resources management system.  **Partial:** The MOH provides some but not all aspects of performance appraisal or career development opportunities, and may receive partner support for IMAM.  **No:** The MOH does not, but partners may provide performance appraisal and career development opportunities for IMAM. |
| Pre-service education | Modules of pre-service education curriculum on comprehensive child health and nutrition | **Full:** The pre-service education curriculum is adapted to include comprehensive child health and nutrition, including the latest evidence on IMAM.  **Partial:** Some but not all aspects of the pre-service education curriculum of child health and nutrition are adapted and updated for IMAM.  **No:** The pre-service education curriculum does not cover IMAM. |
| Continuing professional development | Continuing professional development on comprehensive child health and nutrition | **Full:** The MOH provides continuing professional development on comprehensive child health and nutrition.  **Partial:** The MOH provides continuing professional development on some but not all aspects of child health and nutrition, and may receive partner support for IMAM.  **No:** The MOH does not, but partners may provide continuing professional development for IMAM. |
| **Medical products** |  |  |
| Essential medicines and medical supplies list | List of national essential drugs and medical supplies list, including for IMAM | **Full:** IMAM equipment, drugs and supplies are included in the national essential drugs and medical supplies list.  **Partial:** Some but not all IMAM equipment, drugs and supplies are included in the national essential drugs and medical supplies list.  **No:** None of the IMAM equipment and supplies are included in the national essential drugs and medical supplies list. |
| Procurement system | National drugs and medical supply needs (forecasting and) procurement including for IMAM | **Full:** The MOH (forecasts and) procures drugs and medical supplies that include all IMAM equipment, drugs and supplies.  **Partial:** The MOH (forecasts and) procures some but not all drugs and medical supplies needs for IMAM, and may receive partner support.  **No:** Partners (forecast and) procure all IMAM equipment, drugs and supplies. |
| Logistic management system | National logistic management system for drugs and medical supplies including for IMAM | **Full:** The MOH has logistic management capacities for drugs and medical supplies that include IMAM equipment, drugs and supplies.  **Partial:** The MOH has insufficient logistic management capacities for IMAM equipment, drugs and supplies, and may receive partner support.  **No:** Partners provide all logistic management for IMAM equipment, drugs and supplies. |
| **Service delivery** |  |  |
| Demand generation | Demand generation by activating and informing communities for improved child health and nutrition | **Full:** The MOH manages demand generation activities for improved child health and nutrition including acute malnutrition (to improve service access, use and retention in treatment, and promote social and behaviour change for improved care practices).  **Partial:** The MOH manages some but not all aspects of demand generation activities for improved child health and nutrition including acute malnutrition, and may receive partner support.  **No:** The MOH does not, but partners manage demand generation activities for improved child health and nutrition including acute malnutrition. |
| Early case finding | Early active (by volunteers in the community), systematic (by health workers at the health facility) and enhanced (by carer) case finding for selected child illnesses | **Full:** The MOH provides and supports early active, systematic and enhanced case finding for selected child illnesses including acute malnutrition.  **Partial:** The MOH supports some but not all aspects of early case finding for selected child illnesses including acute malnutrition, and may receive partner support.  **No:** The MOH does not, but partners may provide and support early case finding for acute malnutrition. |
| Community-based primary care | Promotive and preventive community health and nutrition (and community case management) | **Full:** The MOH manages comprehensive community-based primary care (national policy).  **Partial:** The MOH manages some but not all aspects of community-based primary care (national policy) including acute malnutrition, and may receive partner support.  **No:** The MOH does not, but partners may manage some aspects of community-based primary care for acute malnutrition. |
| Outpatient care (facility-based primary care) | Outpatient management of severe acute malnutrition (SAM) without complications and moderate acute malnutrition (MAM) as part of IMCI | **Full:** SAM without complications and MAM are systematically diagnosed and treated with continuity of comprehensive child health as part of IMCI.  **Partial:** SAM without complications and MAM are diagnosed and treated as part of or in isolation from IMCI, and may receive partner support.  **No:** SAM without complications and MAM is diagnosed and treated in isolation from IMCI organised by partners. |
| Inpatient care (child hospital care) | Inpatient management of SAM with complications until stabilisation as part of child hospital care | **Full:** SAM with complications is systematically diagnosed and treated in hospitalised care until stabilisation with the SAM ward (or SAM beds) being part of the paediatric unit with rotating staff.  **Partial:** SAM with complications is diagnosed and treated in hospitalised care until stabilisation or full recovery with the SAM ward separate from the paediatric unit with assigned SAM staff, and may receive partner support.  **No:** SAM with complications is treated until stabilisation or full recovery in a SAM ward separate from the paediatric ward organised by partners. |
| Health outreach | Health outreach activities for selected child illnesses, including acute malnutrition | **Full:** MOH manages comprehensive health outreach activities for selected child illnesses (national policy) including acute malnutrition.  **Partial:** MOH manage some but not all aspects of health outreach activities for selected child illnesses (national policy) including acute malnutrition, and may receive partner support (e.g. for early case finding and referral for treatment).  **No:** MOH does not, but partners may provide health outreach activities for selected child illnesses (national policy) including acute malnutrition (or not). |
| Referral and tracing between services | Referral and tracing system for the detection and retention in treatment of selected child illnesses, including acute malnutrition | **Full:** The MOH manages a referral and tracing system for the detection and retention in treatment of selected child illnesses including acute malnutrition.  **Partial:** The MOH manages some but not all aspects of a referral and tracing system for the detection and retention in treatment including acute malnutrition, and may receive partner support.  **No:** The MOH does not, but partners may organise a referral and tracing system for the detection and retention in treatment of acute malnutrition. |
| Patient-centred continuity of care | Comprehensive child health care tracked over time and place responding to individual preferences, needs and values | **Full:** Comprehensive care for the ill child is provided, tracked over time and place, and responds to the individual preferences, needs and values.  **Partial:** Care for the child with acute malnutrition is being provided in isolation, and may receive partner support for tracking over time and place.  **No:** Care for the child with acute malnutrition is provided in isolation, and is not tracked over time and place. |
| Continuous quality improvement | Continuous quality improvement of comprehensive child health care | **Full:** The MOH manages a continuous quality improvement system of comprehensive child health care by assessing and addressing barriers.  **Partial:** The MOH manages some but not all aspects of quality improvement of child health care including acute malnutrition by assessing and addressing barriers, and may receive partner support.  **No:** The MOH does not, but partners may manage an IMAM-specific quality improvement system. |
| **Health services performance** |  |  |
| Geographic service coverage | Geographic coverage of health facilities with SAM services | Number of sites with SAM services  Proportion (%) of secondary and primary health facilities offering SAM services |
|  | Geographic coverage of health facilities with MAM services | Number of sites with MAM services  Proportion (%) of primary health facilities offering MAM services |
| Access to treatment | Annual number of children under 5 receiving treatment for SAM | Number of children under 5 with SAM receiving treatment |
|  | Annual number of children under 5 receiving treatment for MAM | Number of children under 5 with MAM receiving treatment |
| Quality of care | Annual overall SAM cure, case-fatality and defaulting rates | Low (cure rate ≤75%, case-fatality rate in treatment ≥10% and defaulting rate ≥15%), medium (one or two low scores for cure, case-fatality in treatment or defaulting rate), or high (cure rate >75%, case-fatality rate in treatment <10% and defaulting rate <15%) quality of care. |
|  | Annual overall MAM cure, case-fatality and defaulting rates | Low (cure rate ≤75%, case-fatality rate in treatment ≥10% and defaulting rate ≥15%), medium (one or two low scores for cure, case-fatality in treatment or defaulting rate), or high (cure rate >75%, case-fatality rate in treatment <10% and defaulting rate <15%) quality of care. |
| Contact coverage | Proportion of children under 5 diagnosed with SAM in the population receiving treatment | Low (<30%), medium (30-50%), or high (≥50%) SAM contact coverage. |
| Sustainability | Sustainability based on financial and technical dependence of IMAM interventions | Low (high financial and technical partner dependence), medium (partial financial or technical partner dependence), high (no financial and no technical partner dependence) sustainability.  Proportion (%) of health facilities with IMAM services receive technical partner support; number of technical partners. |
| **Health status** |  |  |
| Prevalence of acute malnutrition | Proportion of children under 5 diagnosed with SAM in the population | Prevalence of children under 5 with SAM at the survey date (%, confidence interval). |
|  | Proportion of children under 5 diagnosed with overall acute malnutrition in the population | Prevalence of children under 5 with overall acute malnutrition at the survey date (%, confidence interval). |
| Under-5 mortality | Probability of dying before 5 years of age, expressed per 1000 live births | Number of children under 5 deaths per 1 000 live births.  (In 2015, the under-5 mortality rate in low-income countries was 76 deaths per 1000 live births – about 11 times the average rate in high-income countries of 7 child deaths per 1000 live births). WHO African Region (81 child deaths per 1000 live births) [1]) |
|  | Proportion of deaths of children under 5 in the population, per 10000 children under 5 per day | Number of deaths of children under 5 per 10000 children under 5 per day prior to the survey date (Assessed in SMART surveys [2]; in emergencies, the rate should not double the baseline rate, or when the baseline rate is unknown or of doubtful validity, the under-5 mortality rate should remain at least below 2.0/10000/day [3]). |

HIS: Health Information System; IMAM: Integrated Management of Acute Malnutrition; IMCI: Integrated Management of Childhood Illness; MAM: Moderate Acute Malnutrition; MOH: Ministry of Health; SAM: Severe Acute Malnutrition.

**References**

1. World Health Organization. 2015. http://www.who.int/gho/child_health/mortality/mortality_under_five_text/en/

2. SMART. Measuring Mortality, Nutritional Status, and Food Security in Crisis Situations: Standardized monitoring and assessment of relief and transitions (SMART) methodology. 2006.

3. The Sphere Project. Humanitarian charter and minimum standards in humanitarian response. Rugby: Practical Action Publishing; 2011.
